# ‘It opens up a whole new world for everybody’: how carers of people with dementia view the online empowered conversations communication course

**DOI:** 10.1080/13607863.2024.2410258

**Published:** 2024-10-09

**Authors:** Cassie Eastham, Yeliz Prior, John Keady, Warren Mansell, Cathy Riley, Mal Walters, Lydia Morris

**Affiliations:** a Greater Manchester Mental Health NHS Foundation Trust, Manchester, UK; b School of Health and Society, University of Salford, Salford, UK; c Division of Nursing, Midwifery and Social Work, University of Manchester, Manchester, UK; d Curtin School of Population Health Curtin University, Perth, Australia; e Open Doors Research Group, Salford, UK; f Division of Psychology, University of Manchester, Manchester, UK

**Keywords:** Caregiver, psychosocial intervention, educational, family, group support

## Abstract

**Objectives:**

This paper explores (1) experiences of participating in Empowered Conversations, an online communication course for carers of people with dementia and (2) how participants felt the course had changed their experience of caring.

**Method:**

Fifteen semi-structured interviews were completed with carers who had attended Empowered Conversations as part of a larger feasibility trial conducted in Greater Manchester, UK (ISRCTN15261686). Data were analysed using applied thematic analysis.

**Results:**

Three themes were developed: (1) You’ve got nothing to lose and everything to gain, including the course content, timing and format, and perceived burden and benefits of the course; (2). A community to share together, including the value of being honest, vulnerable, and sharing stories; and (3) Being given a new way to see the world, including understanding the person and their diagnosis, enabling greater control and reducing interpersonal conflict, and recalibrating their expectations.

**Conclusion:**

Carers reported positive experiences of participating in Empowered Conversations and valued meeting people who, despite different circumstances, shared their understanding of supporting someone with dementia. The course supported participants to be honest and vulnerable, and helped them to rethink communication and have a greater appreciation of the other person’s perspective during everyday interactions.

## Introduction

In the United Kingdom (UK), there are approximately 700,000 people supporting a family member, partner, or friend at home who lives with dementia (Parliament: House of Commons, 2021). Providing unpaid care to a person with dementia can impact on the psychological and physical wellbeing of carers (Carers UK, 2023). Specifically, difficulties with communication are associated with increased carer stress and burden and can contribute to the breakdown of the interpersonal relationship between the carer and person with dementia (Egan et al., 2010).

Changes to language will vary between individuals because of the subtype of dementia, stage of progression, and affected area(s) of the brain, as well as factors influencing the individual’s capabilities to withstand the damage to their brain (‘their brain reserve’) and the effects this has on them (their ‘cognitive reserve’) (Alsawy et al., 2017; Wray, 2020).

Language use impacts on how people experience and understand their social world and as such, everyday conversations are central to the lived experience of people with dementia (Hamilton, 2019; Volkmer et al., 2023). Difficulties communicating are associated with stigma, and loss of self-esteem and individual agency (Hamilton, 2019) and can result in the person with dementia losing confidence, withdrawing during interactions, or feeling disconnected from and devalued by the other person (Alsawy et al., 2017; Braithwaite Stuart et al., 2022; Volkmer et al., 2023).

After diagnosis, carers should be offered psychoeducation and skills-based training that includes a communication component (National Institute for Health & Clinical Excellence, 2018). Multicomponent interventions can improve carers’ levels of depression and potentially can improve mastery, which include self-efficacy, ability, and perceived competence (Cheng & Zhang, 2020). However, due to the heterogeneity of such interventions, there is less evidence identifying which components offer the best outcomes for carers (Carter et al., 2020; Cheng & Zhang, 2020; He et al., 2022).

Interventions that focus on communication can have a positive effect on carers’ knowledge and communication skills (Morris et al., 2018; Nguyen et al., 2018; Perkins et al., 2022) and improve the person with dementia’s communication and behavioural symptoms (Perkins et al., 2022). Communication training has also demonstrated improvement in quality of life for both parties, although evidence for this is weaker (Perkins et al., 2022).

However, communication training programmes often lack a strong theoretical basis (Morris et al., 2018; Perkins et al., 2022), the quality of research is variable (Nguyen et al., 2018; Perkins et al., 2022), and most training focuses on practical communication skills without addressing the emotional and relational contexts of care and communication (Morris et al., 2018; Williams et al., 2018) which are important to people with dementia (Alsawy et al., 2017). Empowered Conversations was developed to in response to these issues.

### What is Empowered Conversations?

Empowered Conversations is a communication course for informal carers of people with dementia that was developed to meet their psychological, relationship and communication needs (Morris et al., 2021). Initially provided as in-person training in a group format, an online version of Empowered Conversations was developed in response to the restrictions during the COVID-19 pandemic when across the UK, dementia support was provided using virtual formats (Wheatley et al., 2022). Although quantitative evidence for the effectiveness of group-based interventions is variable, they are recommended in clinical guidance (National Institute for Health & Clinical Excellence, 2018) and participants report satisfaction with this format, identifying social support and connection as positive factors (Cheng & Zhang, 2020; McLoughlin, 2022). Online interventions, particularly those that are multicomponent, interactive, and offer peer-support have been highlighted as being potentially effective for caregivers but higher quality evidence to is needed to support this assertion (Hopwood et al., 2018; Naunton Morgan et al., 2022).

Empowered Conversations is based on the Communication Empowerment framework, which integrates the concepts of Mentalization Theory, Perceptual Control Theory (PCT), and the Communicative Impact model with the intention of improving communication by utilising the underlying psychological and relational mechanisms (Morris et al., 2020).

PCT proposes that control is fundamental to wellbeing. In this context, control refers to the process of maintaining our experience according to an internal reference value or goal (Powers, 1973). For example, whilst walking, a person controls their posture to keep upright, their speed, and the route they take to avoid obstructions. Control typically runs smoothly unless there is conflict. Conflict is defined as attempting to achieve and maintain a variable at two opposing values. In this example, the goal of moving quickly may conflict with that of dodging obstacles.

Control is also directed towards maintaining abstract variables at a desired state; in everyday life these variables are often known as goals, rules, standards, values, or ideals. Complex human behaviour is possible because the many variables a person controls are organised in a branching hierarchy, where lower-level goals (e.g. completing an exercise class) are a means to achieving and maintaining the higher-level goals (e.g. being healthy). In shared experiences, such as having a conversation, control will switch and balance over time.

Empowered Conversations supports carers to reflect on how control and conflict may be affecting their interactions and provides them with the knowledge and skills to respond differently in these situations. This includes developing mentalization considering the goals of the other person (Morris et al., 2020), as well as considering how they can adapt their own responses and create an environment that allows the person with dementia to have more control during conversations.

More information about Empowered Conversations can be found at www.empowered-conversations.co.uk. Earlier studies have demonstrated that an in-person version of Empowered Conversations is an acceptable intervention, which significantly reduced carer stress and improved communication across time (Innes et al., 2022; Morris et al., 2024). This qualitative study is nested within a feasibility randomised controlled trial (RCT) of an online version of Empowered Conversations (see trial protocol for details (Eastham et al., 2023)). The trial was registered on the ISRCTN platform (ISRCTN15261686).

This study aims to explore (1) carers’ experiences of the online Empowered Conversations course and (2) how they perceive participating in the course has changed their everyday lives.

## Methods

Data was collected using semi-structured interviews and analysed using applied thematic analysis which is appropriate for exploratory studies seeking to address practical problems in real-world settings (Guest et al., 2011). The theoretical approach was critical realist (Creswell, 2009) and therefore it was assumed the language utilised by participants reflected their subjective meaning and experience (Braun and Clarke, 2006).

Groups of 4–10 carers living in Greater Manchester participated in Empowered Conversations using the Zoom platform. During the trial, seven cohorts took part in courses held between June and November 2022; each course consisted of six, two-hour sessions.

Sessions are delivered by two facilitators who come from a range of professional backgrounds, including a physiotherapist, retired general practitioner, and clothing designer, and most of whom have lived experience of being an informal carer of a family member with dementia. A course manual provides a structured framework of core topics, discussions, and activities, with flexibility for the facilitators to adapt the course material to the needs of participants. More information about the course content, delivery, and facilitator training and supervision is available in the RCT protocol (Eastham et al., 2023).

### Participants

Seventy-five participants were recruited to the main RCT and allocated to the treatment and control groups in a 2:1 ratio. During the consent process for the RCT, participants were given the choice to be contacted about completing a qualitative interview. To be eligible for the qualitative study, participants must have been allocated to the treatment group of the RCT and attended three or more sessions. A sample size of fifteen (approximately one-third of eligible participants) was considered to be representative of participants in the full trial (Table 1) and would provide sufficient data for analysis.

**Table 1.** Characteristics of participants in main trial and nested qualitative study.

Demographic characteristic	Main trial *N* (*n* = 75)	Qualitative study *N* (*n* = 15)
Gender		
Male	14 (18.6%)	6 (40%)
Female	59 (78.6%)	9 (60%)
Missing	2 (2.6%)	0
Diagnosis		
Alzheimer’s disease	32 (42.7%)	6 (40%)
Vascular dementia	14 (18.7%)	2 (13.3%)
Mixed dementia	17 (22.7%)	4 (26.7%)
Parkinson’s dementia	1 (1.3%)	0
Frontotemporal dementia	1 (1.3%)	0
Lewy body dementia	3 (4%)	1 (6.7%)
Other type of dementia	5 (6.7%)	2 (13.3%)
Missing	2 (2.7%)	0
Ethnicity		
White	69 (92%)	14 (93.3%)
Mixed/Multiple ethnic groups	1 (1.3%)	0
Asian/Asian British	1 (1.3%)	0
Black/African/Caribbean/Black British	1 (1.3%)	1 (6.7%)
Other ethnic group	1 (1.3%)	0
Missing	2 (2.7%)	0
Relationship		
Spouse/long-term partner	38 (50.7%)	10 (66.7%
Parent	30 (40%)	5 (33.3%)
Sibling	3 (4%)	0
Friend	1 (1.3%)	0
Other relationship	1 (1.3%)	0
Missing	2 (2.7%)	0

Within the main study, all male carers supported their spouse. Female carers supported spouses, parents, and friends or other relatives. Two courses had no male participants.

On completion of their 6-month follow up measures, participants who had given consent to be contacted were emailed with an invitation to take part in an interview. Twenty-one participants were invited and fifteen completed interviews.

Purposive sampling and a sampling matrix were used to ensure that there was representation of carer relationships (partner/spouse or parent/other) and gender. In addition, because each course has a unique group dynamic and experience, at least one participant from each cohort was interviewed.

### Data collection

The interview schedule [Supplementary Info] was based on a previous study of Empowered Conversations (Morris et al., 2021). The topic guide was further developed and finalised with the study team, which included qualitative methodologists, psychologists, mental health nursing and occupational therapists.

Participants were given a choice of formats and venues for the interview and chose; telephone (*n* = 4), Zoom/Teams (*n* = 5), in-person at home (*n* = 4), and in-person at a community venue (*n* = 2). Only the participant and interviewer were present during the interviews.

Interviews lasted between 30 and 83 min, with an average length of 60 min. Most interviews were completed in one appointment, however one required a follow up telephone call because of technical issues with the recording and one was conducted over two telephone calls and email to accommodate the participant’s caring responsibilities. Interviews were audio-recorded using an encrypted Dictaphone or using secure recordings from the online platforms. Notes were made during and after the interviews.

All interviews were conducted by CE who is an occupational therapist with experience of working in National Health Service dementia services and undertaking qualitative interviews. Other than contact during recruitment to the RCT, CE had no prior relationship with the participants.

In line with the study protocol and ethical permission, all identifiable personal information was removed from the data and pseudonyms have been used to report the findings.

### Data analysis

Data were initially coded by CE, with JK and YP independently coding a sample of the transcripts. CE familiarised herself with the data by listening to audio recordings of the interviews, reading and re-reading the interview transcripts, and making notes and highlighting relevant text in the transcripts. Codes were identified and grouped into categories that aligned with the structure of the interview schedule and the aims of the study (Guest et al., 2011). Initially, data were coded manually on hard copies of the transcripts and by using Word and Excel. NVivo was to manage the data at a later stage when themes were more developed.

A coding book (Supplementary Material) was developed by CE and reviewed by the study team and the study’s PPIE group. The group offered feedback about the codes and discussed how these were reflected in their experiences of caring. Three group members of had completed Empowered Conversations and could share their experiences and identify commonalities with the study participants.

Initial themes were then developed by CE and discussed with LM and WM. The feedback advised that the themes should be revised with a renewed focus on the conceptual foundations of Empowered Conversations. The revised themes were reviewed and agreed by LM, WM, JK, YP and CE.

An online member checking session was completed with two participants who both agreed that the themes resonated with their experiences on the course and how they had continued to use and adapt their new approach to communication.

### Public and patient involvement and engagement (PPIE)

A PPIE group for the RCT was set up through the Salford Open Doors research group, a well-established group for people with dementia and current or former carers to become involved to service development and research. The Open Doors Research Group has experience of contributing to dementia studies and provided a valuable perspective on the study’s design and conduct (Keady, 2024). One Open Doors member contributed directly to the qualitative study by reviewing and commenting on interview transcripts and the group was consulted about themes developed from the qualitative interviews and. All PPIE contributors were compensated in keeping with current NIHR policy (National Institute for Health & Care Research, 2022).

### Ethical considerations

The study was reviewed by the Wales Research Ethics Committee 2 and received approval in February 2022 (REC: 22/WA/0010).

## Results

A final set of three themes and nine subthemes was developed (Table 2).

**Table 2.** Final themes and subthemes.

**Theme	Subthemes
You’ve got nothing to lose and everything to gain	Course content and delivery Online experience It’s best early (but not too early)
A community to share together	Having the freedom to be honest and vulnerable Being understood because of what you share People’s stories resonate and educate
Being given a new way to see the world.	Understanding the person through the lens of dementia Recognising control in communication Recalibrating expectations

### Theme 1: You’ve got nothing to lose and everything to gain

Participants gave their views on the experience of the content and delivery of the online version of Empowered Conversations. The theme also demonstrates how participants evaluated the potential burdens and rewards of participating in Empowered Conversations.

#### Course content and delivery

The course provided a combination of information and support, which surprised some participants who had anticipated that the course would be purely educational. However, even those participants for whom the course had been different to their expectations felt that other caregivers would find benefits from taking part.

It will give you an opportunity to think about yourself and your caring situation and it will give you some coping strategies and some communication strategies… [and] it’s an appropriate length of course so you’re not committed for too long. (Yvonne)

Participants felt that the course length was appropriate and that the potential benefits of taking part warranted the investment of their personal resources.

There’s a really strong supportive element to it, there’s a really strong content element that you can refer to… And for six weeks, you’ve got nothing to lose, you’ve got everything to gain. (Martin)

Participants identified different aspects of the course content that they had found interesting, useful, or memorable. These included the exercises that supported them to recognise their own needs as a carer, understand more about dementia, learn communication techniques, and have greater empathy and understanding of the person they support.

The varied content allowed participants to take what they needed from the course, building their own bespoke toolbox of skills to use at the time of participating or in the future.

As things have progressed, I’m beginning to see the benefits of the Empowered Conversations and the need to use strategies, not just for his understanding but for my wellbeing… And then I have to think okay, well what strategies were there that I need to put in place here. So, I’m just beginning to realise just how much I need to revisit and use what you taught us. (Pamela)

Participants valued the facilitators’ warmth, friendliness, and approachability and felt these characteristics created a welcoming and comfortable learning environment. Most Empowered Conversations facilitators have lived experience of caring for someone with dementia, which gave them credibility and authenticity with some participants:

They put you at ease… you know, and they got it, didn’t they? They’ve gone through it themselves, I guess. Yeah, they did put you at ease. (Caroline)

#### Online experience

Participants’ experience of using Zoom or similar applications varied. Participants with less technological experience found the format to be acceptable and described becoming more confident as the course progressed. No major technical issues were reported by the participants and minor problems were solved with the support of the facilitators.

Most participants found the online format acceptable but stated that they would have preferred the course to be delivered in person. These participants associated in-person delivery with potentially improved communication and connection with others, feeling that they could forge stronger relationships and understanding without the barrier of a screen:

I’m very much a face-to-face person. It doesn’t sit comfortably, you can’t pick the nuances up about other people are, and you can’t put an arm around somebody, you can’t be spontaneous, it can’t be iterative… you lose something. (Martin)

However, four participants reported that they had only been able to attend because the course was delivered online and all participants identified at least one advantage to the Zoom format, either in relation to their own situation or from the perspectives of other participants. These benefits related to easier planning and practicalities of delivering the course, for example:

Yeah, the advantages were for anybody who didn’t need to get a carer in. I didn’t have to think about travelling… or parking. So, all those practical things. (Angela)

#### It’s best early (but not too early)

Overall, participants felt that the course would be best delivered during the initial post-diagnostic period, however some participants recognised that this time can be overwhelming and felt that the diagnosis needs time to ‘soak in’. Some participants felt that this would differ between individuals but people needed to be ready to take in information and contribute to the group.

This was reflected by several participants who thought that the timing of Empowered Conversations had not been optimal for their needs:

I think with hindsight if I’d have done it six months earlier, she’d have had the benefits, and I would sooner, because I didn’t realise until I started the course that actually, this had been going on for some time. (Fiona)

However, even when the timing was not right, participants reported benefits from the course, for example, Angela realised that she had not been emotionally ready for the course, but it had helped her with coming to terms with her husband’s diagnosis:

I think it’s helped me slowly start to feel more on a journey towards feeling slightly more comfortable with how things are, and the future now felt a bit more ‘do-able’. (Angela)

### Theme 3: A community to share together

The Empowered Conversations course provided participants with the opportunity to meet other caregivers and experience the support of other people through discussing and sharing experiences and feelings in a supportive environment.

#### Having the freedom to be honest and vulnerable

The support of facilitators and other members of the group gave participants the freedom to be honest about their experiences and to show vulnerability:

Was I comfortable getting upset? No, I wasn’t, but I did get upset and I was upset. But that was a good quality of the group… I didn’t have any reservations of holding back… I knew it would be supportive, it wouldn’t be judging. (Martin)

Participants developed connections and relationships with each other which strengthened as the course progressed. This enabled them to feel more confident in sharing thoughts that were difficult and potentially distressing:

I was just laying it on the table and being quite vulnerable to people who I’d not known for more than six weeks…I think it was well placed where we did that exercise, towards the end of the training, because I think if it was sooner, we wouldn’t have perhaps as felt as comfortable with each other. (Diane)

#### Being understood because of what you share

Participants felt that other people in the group understood them because they were all in similar circumstances. Participants did not have to explain themselves, because other participants had been through the same experiences, or if not the same, they could identify with them through shared understanding:

I almost looked forward to the sessions where you were given the opportunity to [share] because I think it helped establish a connection with the other people. To say, ‘yeah, right, we are all coming from the same direction, we are all in this together’. It’s not as though somebody up there is saying, ‘oh, I don’t understand…why you’re reacting that way, that’s not my experience at all’. (Gary)

This contrasted with participants’ experiences of talking to friends and family:

You can reach out to your family, and you can reach out to your friends, but unless you’re in that journey yourself… unless you’ve gone through it yourself, you can sympathise, you can… show empathy, but you don’t know. (Caroline)

However, if the group had a mix of relationships or ages, some participants found it harder to connect or these differences provoked an emotional reaction in themselves:

I didn’t say anything, but I felt, ‘wait until you’ve got somebody 24 hours a day and you’ve lost your husband, and then tell me’. I know that you can’t split people up, but I did feel there was quite a difference between having somebody…well, having a normal life six days a week, and having just one day a week. (Angela)

#### People’s stories resonate and educate

Participants valued hearing other people’s experiences and seeing their own experiences reflected in the learning materials:

The video clips that we watched were brilliant. All the little Alison Wray films (Wray, 2022) that we looked at, I could see all those scenarios that we were faced with, I could see my mum or myself in those and thinking oh yes, that happened to us. (Fiona)

They reported a sense of relief that these experiences were ‘normal’ and that they were not alone in how they felt:

Just to hear what somebody else is going through, especially if it was a particularly bad week, but it was also good to be able to know that you’re not alone, and it was almost more of a cathartic type of experience. (Diane)

In groups where other participants were caring for someone with more advanced dementia, this could be ‘a double-edged sword’ *(Martin)*. Participants could feel scared thinking about the future, but there could also be a sense of reassurance that other people were coping in that situation and that this was an opportunity to learn from their experiences.

Participants could also recognise how tools and strategies might be useful in the future or had started to use techniques because their situation had changed since completing the course:

As things have progressed, I’m beginning to see the benefits of the Empowered Conversations and the need to use strategies, not just for his understanding but for my wellbeing. (Pamela)

### Theme 4: Being given a new way to see the world

Participants developed a new perspective on the person they support and the interactions within the partnership, whilst also acquiring communication tools and skills. Participants made conscious decisions about how they communicate, instead of defaulting to their previous approach.

Now I’m aware of it, it almost like gives you that little pause to think, ‘hang on a minute, just don’t say that’, whereas before, it just came out, you couldn’t help yourself. (Gary)

#### Understanding the person through the lens of dementia

Participants could identify changes in their communication skills and relate them to understanding more about how dementia can affect a person. This included thinking about how dementia may impact on how the person experiences, understands, and responds to situations. Participants demonstrated curiosity about what the person might be experiencing and applied their understanding to past experiences:

[I] also seemed to get a great understanding to what my wife was probably going through. She couldn’t vocalise a lot of what she was going through… I think a lot of that is because it’s doing her head in this, you know, she can’t see, she doesn’t understand sometimes what’s going on. I think that’s what brings her emotions to the front. (Alan)

This greater understanding also helped caregivers to move negative feelings about the person’s presentation away from the person and on to their dementia:

I think the second thing was, it increased my awareness… in that [it], reminded me that Susan wasn’t well, and it wasn’t her fault. (Martin)

#### Recognising control in communication

Participants became more aware what they could control through their own communication and how they can support the person with dementia to have more control during interactions.

Participants recognised the negative effects that their usual methods of communication could have on the person with dementia and learned how to make changes which gave more positive results. This included noticing how their usual communication methods, such as non-verbal cues, affect the person they support and recognising how the person with dementia interprets their caregiver’s emotional responses:

And I don’t react to that, you know, I just let it wash over as if it’s not happened and we go off talking about something else… I would have reacted before, but I don’t react like that now. So, it must have changed me, yeah, without me realising it. (Alan)

Participants also identified ways in which they could create an environment that supports the person to have more control in the conversation. These included, giving the person more time to respond, not second-guessing what the person wants to say, and using the Invitation to Respond tool. Using Invitation to Respond, participants learn to start a conversation with a statement instead of a question, and then be open and comfortable with wherever this takes the conversation:

All those very, very basic things that you forget because you’re trying to fly in and out of a room and do stuff, so be in front of them, give them time, and make it a conversation that’s more about hopefully them coming alongside you in that discussion rather than a, ‘do you want a cup of tea?’. (Gillian)

#### Recalibrating expectations

Participants learned to adjust their expectations of the person they support within the context of their dementia, for instance, recognising that the person’s ways of communicating and completing tasks have changed. Alongside this, participants could move closer to accepting these changes. This helped them let go of the stress caused by circumstances outside their control and recognise that their stress responses were not helpful:

You know, if it takes him ten minutes to peel a potato, then it takes him ten minutes to peel a potato, but he’s still peeling the potato, that kind of thing. He can still keep some of his independence. And sometimes I have to do the breathing when I can feel I’m getting a bit stressed with him, rather than sort of snapping and saying something I might regret. (Caroline)

Adjusting expectations about the person with dementia sometimes required the participant to make a corresponding adjustment to their perspective on their own identity and role in the relationship:

I feel now that I am the daughter that goes in now and I’m more of a carer. I go to look after this old lady. Because she doesn’t have the capacity to engage in that relationship emotionally that a mother and a daughter have. (Fiona)

However, recalibrating expectations could also result in the participant realising that they had underestimated the person’s level of understanding and communication:

You know I always thought… I thought Wendy doesn’t really understand what I’m doing but she does, obviously that part works still. (Graham)

## Discussion

The study aimed to explore carer’s experiences of participating in the online Empowered Conversations course and participants’ perceptions of changes that occurred because this. The study identified that participants had mostly positive experiences of the content and delivery of an online version of Empowered Conversations. Care partners valued being in a supportive learning environment with their peers and could identify how the course had resulted in changes to themselves, their relationships, and their communication skills.

### Carers’ experiences of empowered conversations

Overall, participants were satisfied with the online version of Empowered Conversations and identified advantages of this format. These included reasons such as not needing to travel, increased convenience, and being more able to manage caring responsibilities at home, which reflect earlier studies (Austrom et al., 2014; Kovaleva et al., 2019; Masoud et al., 2021). However, in keeping with studies of online support groups (Austrom et al., 2014; Banbury et al., 2019), most participants felt that an in-person course would provide a better environment for communicating and connecting with others.

However, participants reported that they did feel connected with other people in the group. This reflects a pilot study of an established psychoeducational programme adapted for online delivery in the United States where participants reported experience of connectedness despite thinking initially that the online format would affect this (Kovaleva et al., 2019). Similarly, in a qualitative study of virtual Memory Cafés in Texas, participants who had only visited an in-person café felt strongly that the online format would not provide a sense of belonging. However, those who had attended an online café reported making meaningful connections with other people (Masoud et al., 2021).

Timing is an important factor for services delivering post-diagnostic support. Offered too early and care partners may not be ready to talk about the diagnosis or accept support, too late and they can struggle to manage their caring role (Boots et al., 2015; Bunn et al., 2012). Generally, participants felt that people in the early stages of caring would benefit from the information and support provided by Empowered Conversations because at this stage they are often making sense of the diagnosis, adjusting to changes in the relationship, and developing strategies to cope with the new situation (Quinn et al., 2008).

However, some participants recognised that the initial post-diagnostic period can be overwhelming both emotionally and in terms of the amount of information that is given at this stage (McCabe et al., 2016). Some participants reported feeling alone after being discharged from diagnostic services and carers can feel that they have been ‘left to get on with it’ (Bamford et al., 2021). Based on the qualitative data within this and the previous study of Empowered Conversations it appears that around six-months to a year after diagnosis could be a particularly useful time to access the course (Morris et al., 2024).

Peer support is established through the commonality of people’s experiences that exists even when there is a breadth of experience in a group (Keyes et al., 2016). However, some participants felt that the experiences of caring within their Empowered Conversations group were too different and that this affected their ability to engage with other people’s contributions. This is consistent with previous studies which reported that differences in severity of dementia, age, and caring relationship could have a negative effect on group participants (Bunn et al., 2012; Kovaleva et al., 2019).

### Perceptions of change

Participants’ perceptions of change are captured in the theme ‘Being given a new way to see the world’ and reflect the theoretical foundations of the Communication Empowerment Framework.

Participants identified how they had modified their communication style to empower the person they support. By making seemingly simple changes, such as using shorter sentences or allowing the person more time to process and respond, the care partner is giving them more control over the conversation and the opportunity to be heard. Similarly, using the ‘invitation to respond’ technique reduces the pressure on the person to provide a ‘correct’ response and allows them to take control of where the conversation might lead (McEvoy et al., 2023).

Consistent with increasing their skills of mentalisation, participants identified that improving their knowledge of dementia and communication helped them to develop a greater understanding of the person they support. Lack of knowledge can cause care partners to attribute dementia-related behaviours to the person rather than their diagnosis, which in turn can cause a strong emotional response such as feeling hurt or angry toward the person (Boots et al., 2015; Stokes et al., 2015). This greater understanding enabled the carer to bring reflection and curiosity to situations, rather than being overwhelmed by dysregulated emotions (Bateman, 2023).

Participants gave examples of the internal goal conflicts they experienced when dementia caused the person to act significantly differently to their pre-morbid personality and abilities. Participants described letting go of their expectations of what the person ‘should’ be able to do, adjusting or abandoning these potentially unattainable goals and replacing them with more realistic ones, which resulted in less conflict and distress for both people (Morris et al., 2020).

Within Empowered Conversations, activities based on PCT support participants to identify what is important to them, within their life and their relationships. It appeared that participants started to reevaluate and reorganise their goals and behaviours according to what they valued most. As such, goals often changed from a low, functional level (for example, wanting the person to load the dishwasher efficiently) to a higher, value-driven level (for example, maintaining the person’s purposeful activities and independence). This enables them to take steps towards addressing their unresolved conflicts by refocussing their attention to the higher level where contradictory goals are established, rather than the lower level where conflicts are presented (Powers et al., 2011).

### Strengths and limitations

Although there was universal acceptance of an online format of EC, the sample is potentially biased as interview participants had been willing to complete the course online. It is important to recognise this because digital inequalities are more likely to affect older people and providing services purely online carries the risk of excluding this group (Chirico et al., 2022). This concern was also raised by the project’s PPIE group.

Although Greater Manchester has a diverse population, this was not represented in the study population, which was predominantly White British and identified as heterosexual. Male carers of people with dementia are often underrepresented in research (Poisson et al., 2023) and one of the strengths of both the main study and the nested qualitative work, is that men were well represented in the sample. There are both service level and research level barriers to minoritised communities accessing dementia services and interventions, future studies should include funding for translation, interpreters, cultural humility training, as well as additional (resourced) outreach into minoritised communities (Brijnath et al., 2022).

Participants in the qualitative study had completed three or more sessions of Empowered Conversations, which may have resulted in a self-selecting sample who had positive experiences. However, 86% of the main study participants (*n* = 65) were adherent to treatment and the average number of sessions attended was 5.08. In a separate project, participants who did not complete the course were approached to take part in an interview about their experience, but there was a low response to this invitation (Dickin 2024).

### Implications for practice and research

Whilst the reported satisfaction with the online format is encouraging, it must be noted that, by joining the trial, participants demonstrated willingness to use the required technology. Future research could offer the choice of in-person or online formats; this would help to reduce the risk of digital exclusion and provide further evidence about each format. Research has also informed updating the course materials to ensure cultural sensitivity (Dowson 2024).

Further investigation into the timing of Empowered Conversations would also be useful to ensure that participants are at an appropriate stage of readiness to participate fully in the course.

## Conclusion

In a landscape where provision of post-diagnostic dementia support is variable or limited (Frost et al., 2021), it appears that Empowered Conversations can offer carers an intervention that is appreciated and accessible. Participants could identify changes in their communication skills, relationship, and role as a carer that had occurred during the course. Improving communication has the potential to improve the lived experiences of both the carer and the person with dementia, however further research is needed to provide evidence around the effectiveness of Empowered Conversations.

## Supplementary Material

Supplemental Material
