# Supplementary material for: ‘It opens up a whole new world for everybody’: how carers of people with dementia view the online empowered conversations communication course
Source: Aging Ment Health. 2024 Oct 9;29(3):558–66. doi: 10.1080/13607863.2024.2410258 (PMC11875430; doi:10.1080/13607863.2024.2410258)
Supplement: Supplemental Material [file CAMH_A_2410258_SM6892.zip › Supplementary_InterviewSchedule.docx]

**Trial of Empowered Conversations dementia carer training
Topic Guide for Semi-Structured Interviews**

*This meeting today is to discuss you how found the Empowered Conversations training course. To help us improve the course and carry out future research, I’ll be asking you questions to find out more about your experience of taking part in both the course and the research project.*

*The first thing that we need to do is complete and sign the consent form (offer to explain this and give participant the opportunity to ask questions).*

*Please keep in mind you can end the interview at any point and we can take a break if you need to. Before we begin have you got any questions?*

***Life before the course*
1. You joined the study about six-months ago, could you tell me about what your life was like at that point?** *Prompt if necessary.*

- *What was going well? What, if anything, was proving to be a challenge?*
- *How would you describe your relationship with NAME at the time of joining the study?*
- *What was your everyday communication like with NAME at this time?*

***Deciding to take part*
2. Can you explain why you decided to take part in this project?**

**3. At the start, what did you hope doing the course would change in your caring life?**

***Experience of the course*
4. Overall, what was the *Empowered Conversations* course like?**

- *What sort of things did you do? > How was that?*
- *Can you give me an example of something new that you learned and applied to your everyday caring?*
- *Did you connect with the other carers doing the course? Did you learn anything from their experiences?*
- *What did you enjoy? / What didn’t you like?*
- *What would you like to change about the course?*
- *If you were to tell another carer about Empowered Conversations, what would you say?*

*What was your experience of using technology such as Zoom/video calling before the course? Did you need support to access this during the course?*

**5. I’d like to know about what you think about the course being online…**

- *How would you describe your relationship with technology?*
- *Did you have any concerns about the course being online? What were they?*
- *Advantages/disadvantages?*
- *Did anything go wrong? Tell me about that…*
- *Was anything helpful about doing it online? Tell me about that…*

***Life Now*
6. It’s been a few months since you did the course, how are things now?**

- *What’s different? Is anything better/worse?*
- *How has your relationship/communication/have you, changed?*
- *Have any changes kept going since you finished the course?*
- *Can you give me an example of something that you do differently now?*
- *Have you told other people about doing the course?*

**Being Part of a Research Study
The course was run as part of a research study, I’d like to ask some quick questions about taking part in research**

*The research part of what you’ve done includes finding out about the study and deciding to take part, doing the questionnaires at the start of the trial and 6-months later, possibly having a visit to speak with the person you support, and this interview.*

*I’ve brought these just to remind you about these things (PIS/Advert/Brief Info/CDR/CRF).*

1. **How did you find taking part in this study?**
   Is there anything you would like to change about how the study is delivered?
2. **Empowered Conversations is open to people who are starting out on their journey as a carer or might have spent a lot longer caring for someone with dementia.**

**What do you think would be the best time for someone to be offered the course?**

*Was this the right time for you to do the course? If no, why was that?*

1. **There was a 6-month gap between doing the questionnaires at the start of the study and doing then at 6-month follow-up.
   Did you still feel part of the project during that time? Did that matter to you?**

- What could the team do to make someone feel more engaged during that time?
- Was there anything that would have been helpful to know between the course and the follow-up questionnaires.

1. **The questionnaires asked you about being a carer, your relationship with the person you support and your mental and physical wellbeing (*use visual prompt*).
   Which of these was the most important to you?**
   - (*If none*) Is there something else that would have been important.
2. **Is there anything else that you would like to tell me about taking part in the study?**

**Thank you for taking part in the interview today. Before we finish is there anything else that you would like to add?**

**Visual aid for Q10**

**My emotions and how I feel**

**My levels of stress**

**My own physical health**

**The support I have as a carer**

**How I feel about being a carer**

**How I feel about the future**

**My relationship with the person I care for**

**My own mental health**

**How I communicate with the person I care for**

**How often I use health services**
