# Supplementary material for: ‘It opens up a whole new world for everybody’: how carers of people with dementia view the online empowered conversations communication course
Source: Aging Ment Health. 2024 Oct 9;29(3):558–66. doi: 10.1080/13607863.2024.2410258 (PMC11875430; doi:10.1080/13607863.2024.2410258)
Supplement: Supplemental Material [file CAMH_A_2410258_SM6892.zip › Supplementary_ThematicAnalysis.docx]

**Development of thematic analysis**

| **EC Category** | **TFA Construct** | | | | | | | | | | |
| --- | --- | --- | --- | --- | --- | --- | --- | --- | --- | --- | --- |
|  | **Ethicality** | **Affective Attitude** | | **Burden** | | **Opportunity Costs** | | **Perceived Effectiveness** | | **Self-efficacy** | **Intervention Coherence** |
|  |  | *Anticipated* | *Experienced* | *Anticipated* | *Experienced* | *Anticipated* | *Experienced* | *Anticipated* | *Experienced* |  |  |
| **Expectations / Context** | **X** | **X** |  | **X** |  | **X** |  | **X** |  | **X** |  |
| **Course content** |  |  | **X** |  |  |  |  |  | **X** |  | **X** |
| **Learning** |  |  |  |  |  |  |  |  | **X** |  | **X** |
| **Experiences** |  |  | **X** |  | **X** |  | **X** |  | **X** |  |  |
| **Online** | **X** | **X** | **X** | **X** | **X** |  |  | **X** | **X** | **X** |  |
| **Now** |  |  |  |  |  |  |  |  | **X** | **X** | **X** |

*Mapping Empowered Conversations coding to TFA constructs*

| **Course Content** | **Experiences** | **Online** | **Learning** | **Now** |
| --- | --- | --- | --- | --- |
| Activity: Videos | Delivery | Experiences with technology | Learning to do things differently | Gaining and using knowledge |
| Activity: “Fried Egg” exercise | Facilitators | Technical issues | Learning to understand both the person and dementia | Doing things differently |
| Activity: Bookcase analogy | Length of sessions | Personal preferences |  | Stopping and changing direction |
| Activity: Self-care | Pressing pause on life | Connection and communication |  | Change within the carer |
| Activity: Other | Being part of a group | Planning and practicalities |  | Continuing to look in the toolbox |
|  |  |  |  | Challenges to using Empowered Conversations |

*Initial categories and codes within each category*

| **Learning**  *Definition: The participant discusses what they learned during the Empowered Conversations course.* | | |
| --- | --- | --- |
| **Code** | **Definition** | **Example** |
| Learning to do things differently | The participant describes how the course has changed how they respond to situations involving their care partner, through their own behaviour/responses/communication. | Can you imagine being told that your father's dead every single day?... because that's what it's like…So, you've just got to think of that and think what you're doing. (Stephen) |
| Learning to understand both the person and the dementia | The participant demonstrates acquiring knowledge about how the person’s dementia affects their behaviour / responses / communication (includes the idea of “putting yourself in their shoes”) | To literally put myself in his shoes on occasions during these sessions so that I could see the world from his perspective. And to make sure that my interactions with him…he would see it in a better light as I started to learn better ways of how to communicate with him. (Diane) |

*Example of category “Learning” with code definitions and examples.*
